# Chlorophyll content estimation in an open-canopy conifer forest with Sentinel-2A and hyperspectral imagery in the context of forest decline

**DOI:** 10.1016/j.rse.2019.01.031

**Published:** 2019-03-15

**Authors:** P.J. Zarco-Tejada, A. Hornero, P.S.A. Beck, T. Kattenborn, P. Kempeneers, R. Hernández-Clemente

**Affiliations:** a European Commission (EC), Joint Research Centre (JRC), Via E. Fermi 2749 – TP 261, 26a/043, I-21027 Ispra, VA, Italy; b Instituto de Agricultura Sostenible (IAS), Consejo Superior de Investigaciones Científicas (CSIC), Alameda del Obispo s/n, 14004 Cordoba, Spain; c Department of Geography, Swansea University, SA2 8PP Swansea, United Kingdom; d Institute of Geography and Geoecology, Karlsruhe Institute of Technology (KIT), Kaiserstraße 12, 76131 Karlsruhe, Germany

**Keywords:** Sentinel-2A, Red edge, Hyperspectral, Forest decline, Chlorophyll, Radiative transfer

## Abstract

With the advent of Sentinel-2, it is now possible to generate large-scale chlorophyll content maps with unprecedented spatial and temporal resolution, suitable for monitoring ecological processes such as vegetative stress and/or decline. However methodological gaps exist for adapting this technology to heterogeneous natural vegetation and for transferring it among vegetation species or plan functional types. In this study, we investigated the use of Sentinel-2A imagery for estimating needle chlorophyll (C_a+b_) in a sparse pine forest undergoing significant needle loss and tree mortality. Sentinel-2A scenes were acquired under two extreme viewing geometries (June vs. December 2016) coincident with the acquisition of high-spatial resolution hyperspectral imagery, and field measurements of needle chlorophyll content and crown leaf area index. Using the high-resolution hyperspectral scenes acquired over 61 validation sites we found the CI chlorophyll index R_750_/R_710_ and Macc index (which uses spectral bands centered at 680 nm, 710 nm and 780 nm) had the strongest relationship with needle chlorophyll content from individual tree crowns (r^2^ = 0.61 and r^2^ = 0.59, respectively; *p* < 0.001), while TCARI and TCARI/OSAVI, originally designed for uniform agricultural canopies, did not perform as well (r^2^ = 0.21 and r^2^ = 0.01, respectively). Using lower-resolution Sentinel-2A data validated against hyperspectral estimates and ground truth needle chlorophyll content, the red-edge index CI and the Sentinel-specific chlorophyll indices CI-Gitelson, NDRE1 and NDRE2 had the highest accuracy (with r^2^ values >0.7 for June and >0.4 for December; *p* < 0.001). The retrieval of needle chlorophyll content from the entire Sentinel-2A bandset using the radiative transfer model INFORM yielded r^2^ = 0.71 (RMSE = 8.1 μg/cm^2^) for June, r^2^ = 0.42 (RMSE = 12.2 μg/cm^2^) for December, and r^2^ = 0.6 (RMSE = 10.5 μg/cm^2^) as overall performance using the June and December datasets together. This study demonstrates the retrieval of leaf C_a+b_ with Sentinel-2A imagery by red-edge indices and by an inversion method based on a hybrid canopy reflectance model that accounts for tree density, background and shadow components common in sparse forest canopies.

## Highlights

• Chlorophyll indices and model inversion methods assessed with Sentinel-2A data.
• Chlorophyll content estimation conducted on open-canopy conifer forest.
• Sentinel-2A chlorophyll indices were successful but showed non-linear trends.
• Chlorophyll estimation from Sentinel-2A with INFORM yielded r^2^ = 0.71.
• Without red-edge bands performance decreased (r^2^ = 0.44/RMSE = 22.8 μg/cm^2^).

## 1 Introduction

Slow-acting disturbance processes, including droughts and pathogen outbreaks, appear to be increasing in various forest ecosystems (Hartmann et al., 2018). This trend may become clearer and stronger as further climate change is likely to exacerbate droughts (Trenberth et al., 2014), cause new biotic disturbance regimes, while it shifts the suitable habitats of many tree species geographically (Allen et al., 2015; Gauthier et al., 2015; Millar and Stephenson, 2015). Early detection of decreasing vitality of dominant forest tree species, i.e. of forest decline, may reveal such disturbances and is critical to assess or mitigate their impacts before more profound structural or compositional changes occur (Kautz et al., 2017; Trumbore et al., 2015). Chlorophyll content (C_a+b_) is an important indicator of plant photosynthetic status. As such, reductions in chlorophyll content may be useful for detecting vegetative decline (Allen et al., 2010; Hoshizaki et al., 1988). In fact, chlorosis is one of the most widely used parameters to monitor decline processes in forestry (Granke and Mues, 2010). Additionally, nitrogen is a key element of chlorophyll, and suboptimal nitrogen and chlorophyll levels affect photosynthesis (Evans, 1989). As a result, chlorophyll content has been proposed as a proxy for nitrogen status in several studies due to its critical role in agriculture and global carbon cycle (Baret et al., 2007; Oppelt, 2002; Oppelt and Mauser, 2004; Vos and Bom, 1993; Yoder and Pettigrew-Crosby, 1995). Canopy chlorophyll *a* + *b* content is itself related to productivity, which among other parameters such as leaf area index (LAI), leaf fraction exposed to light, fractional cover, biomass and absorbed photosynthetic active radiation, are critical to understand plant functioning (Clevers and Gitelson, 2013) and to model the earth's biogeochemical system.

Phenology responses in pigment content and leaf area index exhibit distinct seasonal variations depending on the species (Gamon et al., 2016; Gond et al., 1999) and level of decline (Ciesla et al., 1994; Hernández-Clemente et al., 2011). In evergreen conifers, chlorophyll and carotenoid concentration significantly increase during the growing season (Porcar-Castell et al., 2008), this increase being higher for healthy trees than for declining trees (Zarco-Tejada et al., 2018). Hence, the increase in the photosynthetic activity during the growing season and the performance of trees with a different health condition may have important implications for the estimation of chlorophyll content at different times of the year.

The retrieval of chlorophyll content via non-destructive remote sensing methods has been the focus of several previous studies which have demonstrated its absorption effects in the red-edge and green spectral regions (Carter, 1994; Gitelson and Merzlyak, 1996; Rock et al., 1988; Vogelmann, 1993). In particular, it has been demonstrated that the red-edge region is highly sensitive to C_a+b_ while largely unaffected by other plant structural properties (Horler et al., 1983). Due to the complexity of the radiative transfer at the canopy level, the estimation of chlorophyll content has typically been carried out by combining narrow-band indices and spectral and derivative ratios (a full review of indices can be found in Haboudane et al., 2004, Haboudane et al., 2002; Zarco-Tejada et al., 2001, Zarco-Tejada et al., 2005). In the context of agriculture, normalizing indices such as the Transformed Chlorophyll Absorption in Reflectance Index, TCARI (Haboudane et al., 2002) or the Modified Chlorophyll Absorption in Reflectance Index, MCARI (Daughtry et al., 2000) with the Optimized Soil-Adjusted Vegetation Index, OSAVI (Rondeaux et al., 1996) (e.g. TCARI/OSAVI and MCARI/OSAVI), has been shown to minimize effects of the soil background and scattering processes caused by structural canopy properties. An alternative approach, combining narrow-band hyperspectral indices with radiative transfer modelling has been successful at predicting C_a+b_ for uniform crops (Haboudane et al., 2002), as well as orchards planted in grids (Zarco-Tejada et al., 2004a), yielding errors below 10 μg/cm^2^.

Due to the spatial complexity of natural vegetation relative to uniform agricultural fields, estimation of C_a+b_ via spectra in the red-edge region is not straightforward. The red-edge region has been successfully used to estimate C_a+b_ in closed forest canopies (Hernández-Clemente et al., 2017) and conifer forests (Zarco-Tejada et al., 2004b) due to the resistance of the red-edge region to crown shadows (Curran et al., 1990; Hernández-Clemente et al., 2012; Moorthy et al., 2008; Zarco-Tejada et al., 2001). In such closed forest canopies, the structure, shadows and background have limited effects on reflectance, yielding reasonable accuracies (<15 μg/cm^2^). In open canopies, direct understory effects as well as between-crown shadows, make the red-edge region incapable of predicting C_a+b_ accurately (Meggio et al., 2010).

For open crop canopies, C_a+b_ has been successfully estimated using red-edge indices scaled up through radiative transfer canopy-level models such as SAILH (Verhoef, 1984) and the Forest Light Interaction Model (FLIM) (Rosema et al., 1992) and later integrated in the Invertible Forest Reflectance Model INFORM (Atzberger, 2000; Schlerf and Atzberger, 2006). These models quantify stand reflectance considering the crown transparency effects on shadowed soil background. More complex approximations accounting for forest structure using 3-D canopy models have also been used (Gastellu-Etchegorry et al., 1996; Goel and Thompson, 2000; Li et al., 1995; Myneni et al., 1991; North, 1996) by coupling a 3-D canopy model with the leaf radiative transfer model PROSPECT and red-edge indices. Models such as DART (Gastellu-Etchegorry et al., 1996), 4-Scale (Chen et al., 1997), GORT (Li et al., 1995) and FLIGHT (North, 1996) have pioneered this approach. However, the large number of parameters needed to adapt the 3-D models can limit inversion procedures (Banskota et al., 2015; Hernández-Clemente et al., 2014; Malenovský et al., 2013; Oliveira et al., 2017; Yáñez-Rausell et al., 2015).

The launch of Sentinel-2A in 2015 and Sentinel-2B in 2017 potentially enables the estimation of chlorophyll content in vegetation (Clevers and Gitelson, 2013) and other biophysical parameters (Delegido et al., 2011; Frampton et al., 2013) at an unprecedented spatial (10 m/20 m) and temporal resolution (10 days at the equator with one satellite, and 5 days with satellites 2A and 2B). The MultiSpectral Imager (MSI) on board Sentinel-2 has two spectral bands in the red-edge region which are theoretically sensitive to C_a+b_, allowing its operational estimation. Previous satellite sensors with red-edge bands include MERIS (Clevers et al., 2001) at 300 m spatial resolution, and Hyperion and Chris-Proba at 30 m spatial resolution. In the case of MERIS on board ENVISAT, the red-edge region was exploited to retrieve biochemical and biophysical parameters for forest monitoring (Hu et al., 2008), although at a much coarser spatial resolution than is possible with Sentinel-2.

Previous studies have used Sentinel-2-simulated data from existing airborne sensors (i.e. APEX used to simulate Sentinel-2) (Laurent et al., 2014) to estimate C_a+b_ retrieval capabilities for grasses (Clevers and Gitelson, 2013) and maize (Schlemmer et al., 2013) and from multi-site campaigns (Frampton et al., 2013) using machine learning algorithms (Verrelst et al., 2012), assessing the uncertainty on the retrievals (Verrelst et al., 2013). The inversion of radiative transfer models for LAI estimation from Sentinel-simulated data (Atzberger and Richter, 2012), and the assessment of the ill-posedness effects on the inversion methods (Zurita-Milla et al., 2015) demonstrate the wide interest in the biochemical and biophysical retrievals from the Sentinel-2 bandset using physically-based approaches. However, most of these studies have been theoretical, using simulated data for closed agricultural canopies. It is unclear how these methods perform for sparse and open forest canopies since these indices and model-inversion methods can be distorted by shadows and understory in the 10–20 m Sentinel-2 pixels. Additionally, to the best of our knowledge, no studies have validated the retrieval of C_a+b_ using real Sentinel-2 imagery and field validation data in heterogeneous coniferous stands.

In this study, we evaluated the estimation of needle C_a+b_ for a sparse pine forest undergoing decline using Sentinel-2A imagery from two different phenological stages (summer and winter). Field measurements of needle C_a+b_ and hyperspectral imagery collected at 40 cm resolution concurrently with the Sentinel-2A overpasses were used to validate two general estimation methods: i) Sentinel-2 indices related to C_a+b_, and ii) an inversion method using the hybrid INFORM model. Using high-spatial resolution hyperspectral imagery we quantified the proportion of scene components (crown, understory, shade) at the Sentinel-2A pixel resolution to assess the effects of shadows and background on the needle C_a+b_ estimates.

## 2 Materials and methods

### 2.1 Study region & field data collection

**Fig. 1:**
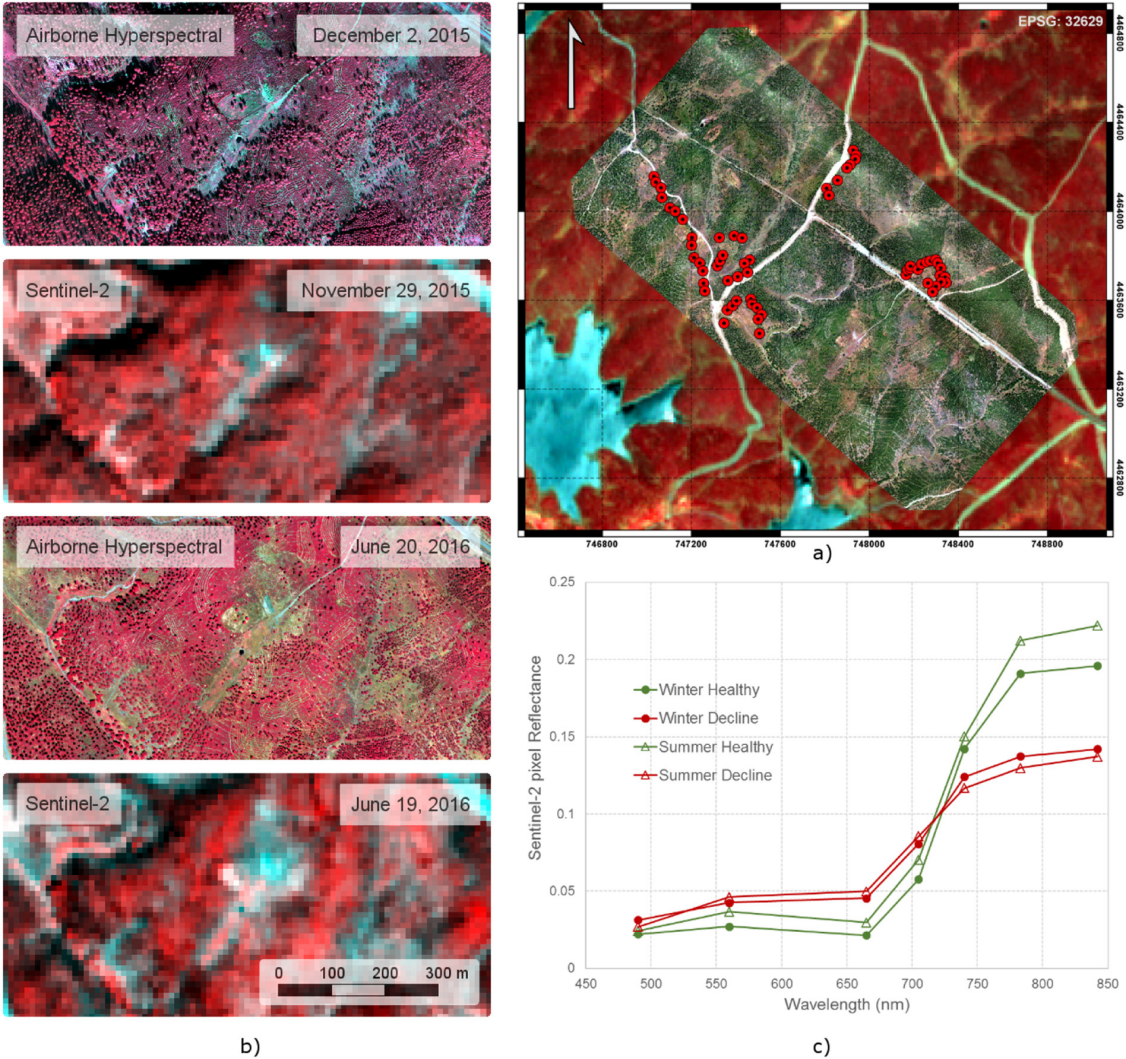
Sentinel-2A scene with hyperspectral image overlay showing the study sites used for validation (a); a zoom over a smaller area (b) of the high-spatial resolution hyperspectral image and the Sentinel-2A scene for June and December acquisition dates. The Sentinel-2A spectral signature of pixels representing healthy and declining trees for both dates is shown in (c).

Analyses were conducted for 61 pine-forest sites undergoing forest decline within the Extremadura region of Spain (40°18′ N, 6°6′ W, 370 to 1000 m.a.s.l.) (Fig. 1). The sites were dominated by *Pinus pinaster*, with *Pinus nigra* mostly occurring at higher elevations. Recently, a general decline has appeared in *Pinus pinaster* in this area (Arzac et al., 2018; Vega et al., 2011). The gradient of mortality indicates damages caused by biotic and abiotic factors. Namely, the decline is intensified with predisposing factors such as water stress or forest health condition affected by ophiostomatoid or other fungi such as *Diplodia pinea* (Matusick et al., 2010; Prieto-Recio et al., 2015). Symptoms of decline included canopy defoliation, discoloration (i.e. chlorosis) and die-off resulting in exposed branches and shoots. Local authorities assessed forest condition between 2014 and 2016, visiting a network of individual trees at each site and scoring them for levels of defoliation, discoloration and canopy die-off. For this particular study, the selected network of sites covered a wide gradient of chlorophyll content. Tree selection was carried out by visual inspection of defoliation and discoloration status of the tree crowns.

Leaf biochemical constituents measured from selected trees at each of the 61 study sites were the chlorophyll (C_a+b_) and carotenoid (C_x+c_) content. Leaf pigment content was measured by destructive methods on 915 needles from 5 samples of 3 needles per crown. We selected needles from the top of the crown in four directions representing the fraction of crown visible by the sensor following the methodology validated by Hernandez-Clemente et al. (2014). Immediately after sampling, needles were frozen in liquid nitrogen in the field and kept under −20 °C until total chlorophyll (C_a+b_) and total carotenoid (C_x+c_) determinations were conducted one week later. Pigment extracts were obtained from a 2 cm^2^ mixture of ground needle material per sample as in Moorthy et al. (2008). The needles were ground in a mortar on ice with liquid nitrogen and diluted in acetone up to 5 ml (in the presence of Na ascorbate). Extracts were then filtered through a 0.45-μm filter to separate the pigment extracts from the Na ascorbate. Absorption at 470, 644.8 and 661.6 nm was measured with the spectrophotometer to derive chlorophyll *a* and *b*, and total carotenoid concentrations (Abadía and Abadía, 1993). The extractions and measurements were undertaken concurrently to avoid pigment degradation.

In July 2016 the following measurements were recorded for each tree: trunk diameter at 1.3 m, total tree height, crown diameter, crown height and crown LAI. LAI was measured using the LAI-2000 Plant Canopy Analyzer (LI-COR, Inc., Lincoln, NE), positioning the optical sensor in eight different orientations under the canopy, at 1 m distance from the ground. A cup which covered 90° of the field of view affected by the trunk was used. Measurements required for LAI estimation included a reference reading above the canopy and below-canopy readings. All measurements were taken before sunrise, after sunset, or under a uniformly overcast sky. Tree coordinates were logged using a GPS device (GPSMAP 60CSx, Garmin International, Inc.) with a spatial accuracy below 2 m.

### 2.2 Sentinel-2A and airborne hyperspectral imagery

Scenes from the MultiSpectral Imager (MSI) on board Sentinel-2A were used for this study. The MSI acquires imagery at ten-day intervals at the equator under constant viewing conditions (Fig. 1a). The images are acquired at 12 bits in 13 spectral bands at different spatial resolutions: four bands at 10 m spatial resolution (central wavelengths at 496.6, 560.0, 664.5 and 835.1 nm with a bandwidth of 98, 45, 38 and 145 nm, respectively), six bands at 20 m (central wavelengths at 703.9, 740.2, 782.5, 864.8, 1613.7 and 2202.4 nm with a bandwidth of 19, 18, 28, 33, 143 and 242 nm, respectively) and three bands at 60 m (central wavelengths at 443.9, 945.0 and 1373.5 nm with a bandwidth of 27, 26 and 75 nm, respectively). The images acquired over the study area were obtained on November 29th 2015 and on June 19th 2016. They were atmospherically corrected from Top-Of-Atmosphere (TOA) Level-1C to generate Level-2A with Sen2Cor (version 2.3.1) on the Joint Earth Observation Data Processing Platform (JEODPP) (Soille et al., 2018) within the Joint Research Centre (JRC) of the European Commission (EC). The processing chain from Level-0 to Level-1C was carried out by the Instrument Data Processing (IDP) functionality of the Payload Data Ground Segment (PDGS).

**Fig. 2:**
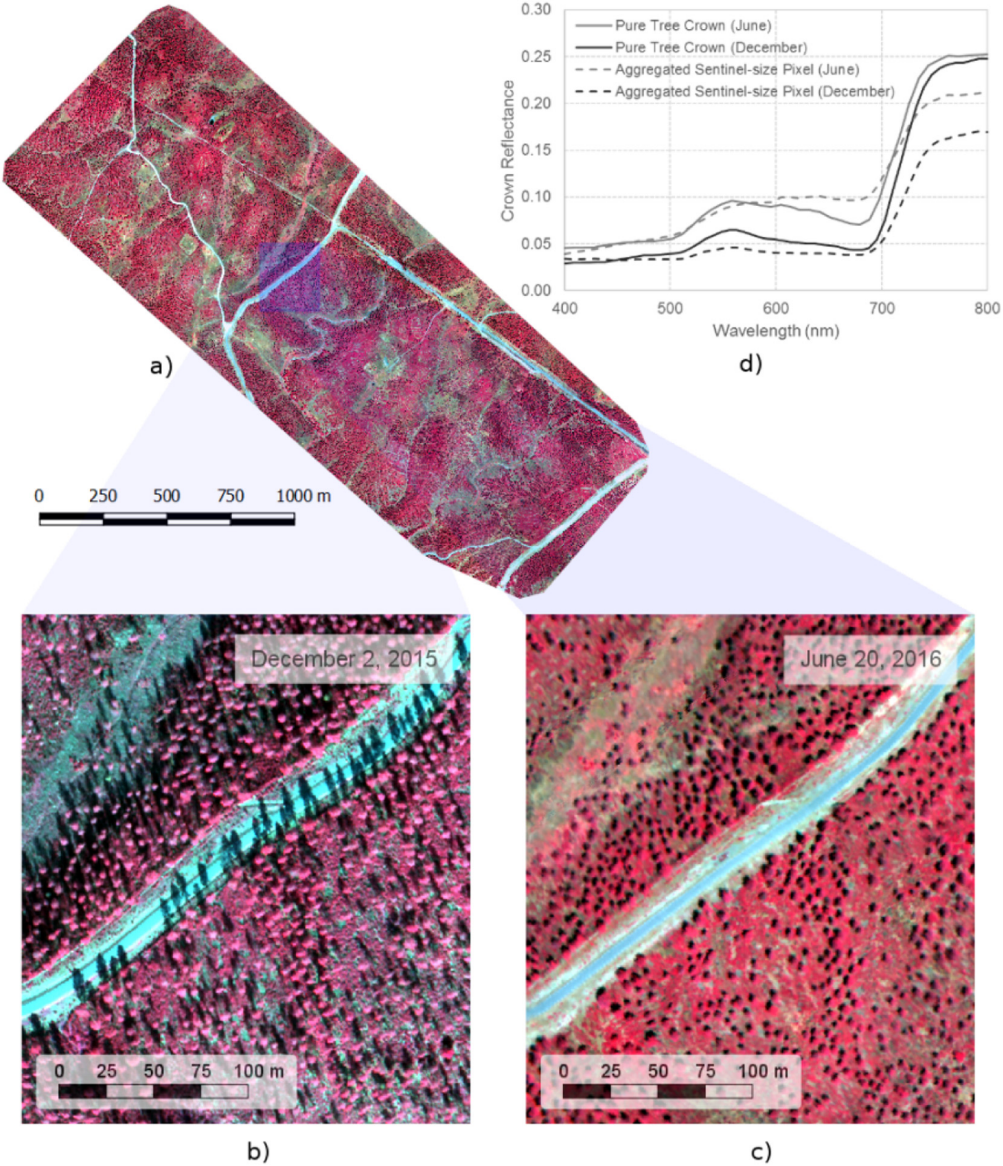
Airborne hyperspectral image acquired with the VNIR hyperspectral sensor at 40 cm resolution over the entire area of study (a) showing the large shadow effects due to sun-angle changes between December (b) and June (c) acquisition dates. The hyperspectral reflectance of a pure tree crown, and the corresponding Sentinel-2A aggregated pixel, show larger shadow effects in the aggregated pixel than in the pure tree reflectance as a function of the acquisition date (d).

For validation purposes, two airborne campaigns were conducted on December 2nd 2015 and June 20th 2016 using a VNIR micro-hyperspectral imager A-series (Headwall Photonics, Fitchburg, MA, USA) on board a Cessna aircraft operated by the Laboratory for Research Methods in Quantitative Remote Sensing, QuantaLab, Consejo Superior de Investigaciones Científicas (IAS-CSIC, Spain) (Fig. 2). The images were acquired flying with the heading on the solar plane at 400 m above ground level at 12:00 GMT yielding a swath of 380 m at 40 cm pixel resolution. The camera was set to 50 fps with an integration time of 18 ms, using an 8 mm focal length lens to yield an instantaneous field of view (IFOV) of 0.93 mrad and an angular field of view (FOV) of 50°. The images were collected in the 400–885 nm region with 260 bands at 1.85 nm/pixel and 12-bit resolution, yielding a 6.4 nm full-width at half-maximum (FWHM) with a 25-micron slit. The flight lines acquired by the hyperspectral sensor were orthorectified and radiometrically calibrated as in Zarco-Tejada et al. (2016) to convert the radiance values to reflectance using the Simple Model of Atmospheric Radiative Transfer of Sunshine (SMARTS) model (Gueymard, 1995, Gueymard, 2005, Gueymard, 2001; Gueymard et al., 2002) with aerosol optical depth measured in the field at 550 nm with a Micro-Tops II sunphotometer (Solar LIGHT Co., Philadelphia, PA, USA). This model was successfully applied in previous studies (Berni et al., 2009; Calderón et al., 2013; Calderón et al., 2015; Zarco-Tejada et al., 2012).

The hyperspectral and the Sentinel-2A datasets acquired on each date were co-registered to ensure the highest spatial agreement, and to minimize geometric errors typically found in high-spatial resolution hyperspectral mosaics due to the multiple flight lines. Additionally, the hyperspectral mosaics were resampled to the Sentinel-2A spectral bandset and spatial resolution, to be used later for the assessments between datasets and for simulation using the radiative transfer models. The pixel aggregate method was used to resize the spatial component, which averages the pixel values gathered by the hyperspectral imager contributing to the simulated Sentinel-2A output pixel; in addition, spectral resampling was performed to match the corresponding FWHM and centre wavelength (CWL) of each Sentinel-2A band. Both Sentinel-2A and the hyperspectral datasets displayed the expected seasonal and sun angle differences between December and June (Fig. 1b) which were observable in the Sentinel-2A VNIR reflectance spectra extracted for a healthy site and a site in decline (Fig. 1c).

The high-spatial resolution imagery acquired with the hyperspectral sensor (ground sampling distance 40 cm) enabled the simulation of pixel aggregation at the Sentinel-2A resolution, as well as the identification of individual scene components (i.e., individual tree crowns, crown shadows and understory) (Fig. 2b). Scene components extracted from the hyperspectral imagery for each Sentinel-2A simulated pixel were used later for radiative transfer modelling. The seasonal variation in shadows between December (Fig. 2c) and June (Fig. 2d) resulted in different aggregation effects at the Sentinel-2A pixel-level, emphasizing the need for this component level information for accurately estimating C_a+b_.

### 2.3 Image segmentation and classification of the hyperspectral imagery

**Fig. 3:**
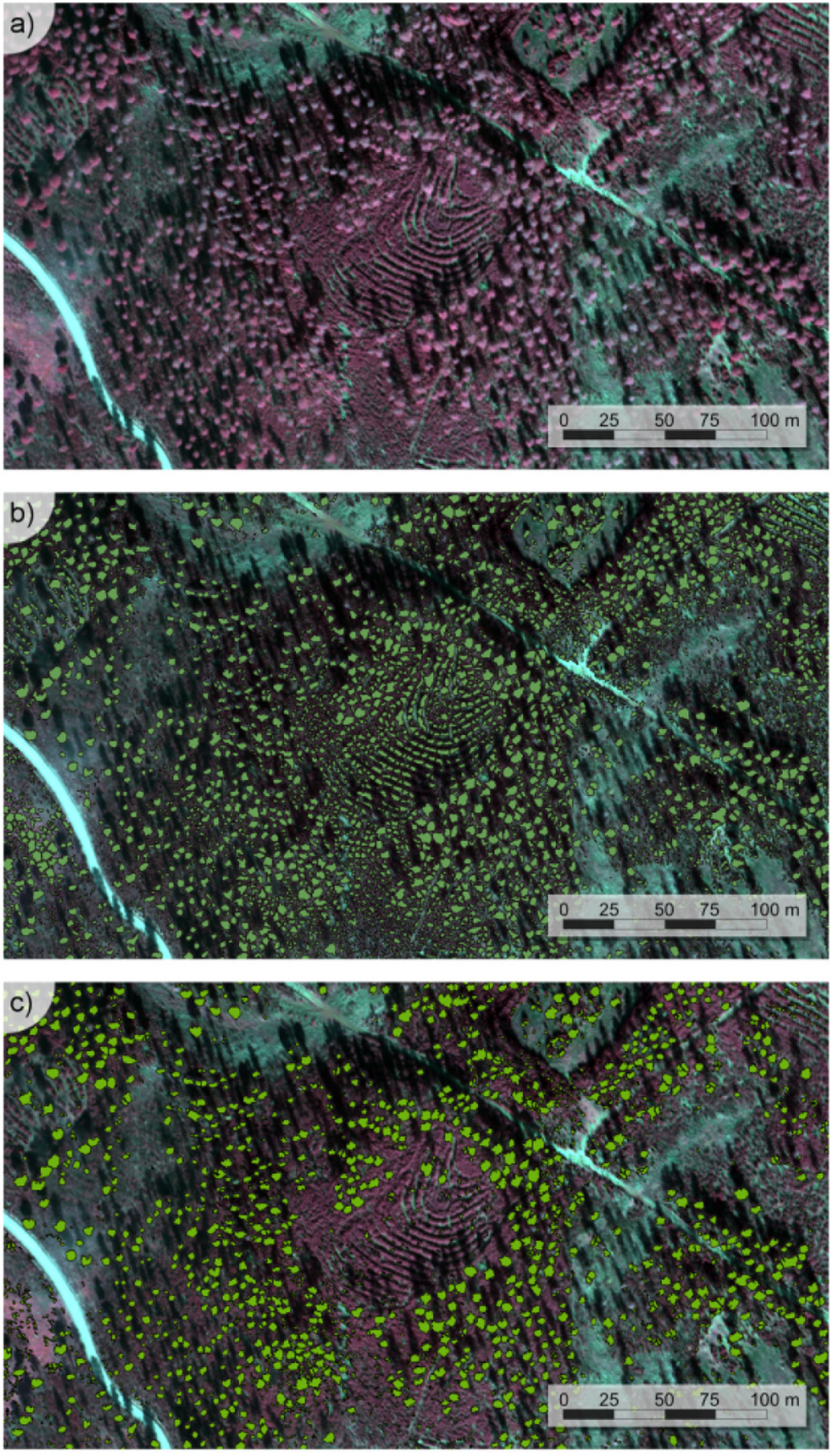
Image segmentation results separating crowns and understory from the shadow and sunlit soil components, showing the original hyperspectral image (a), the segmentation of the tree crowns and understory (b), and the selection of pure tree crowns without the understory component (c).

**Fig. 4:**
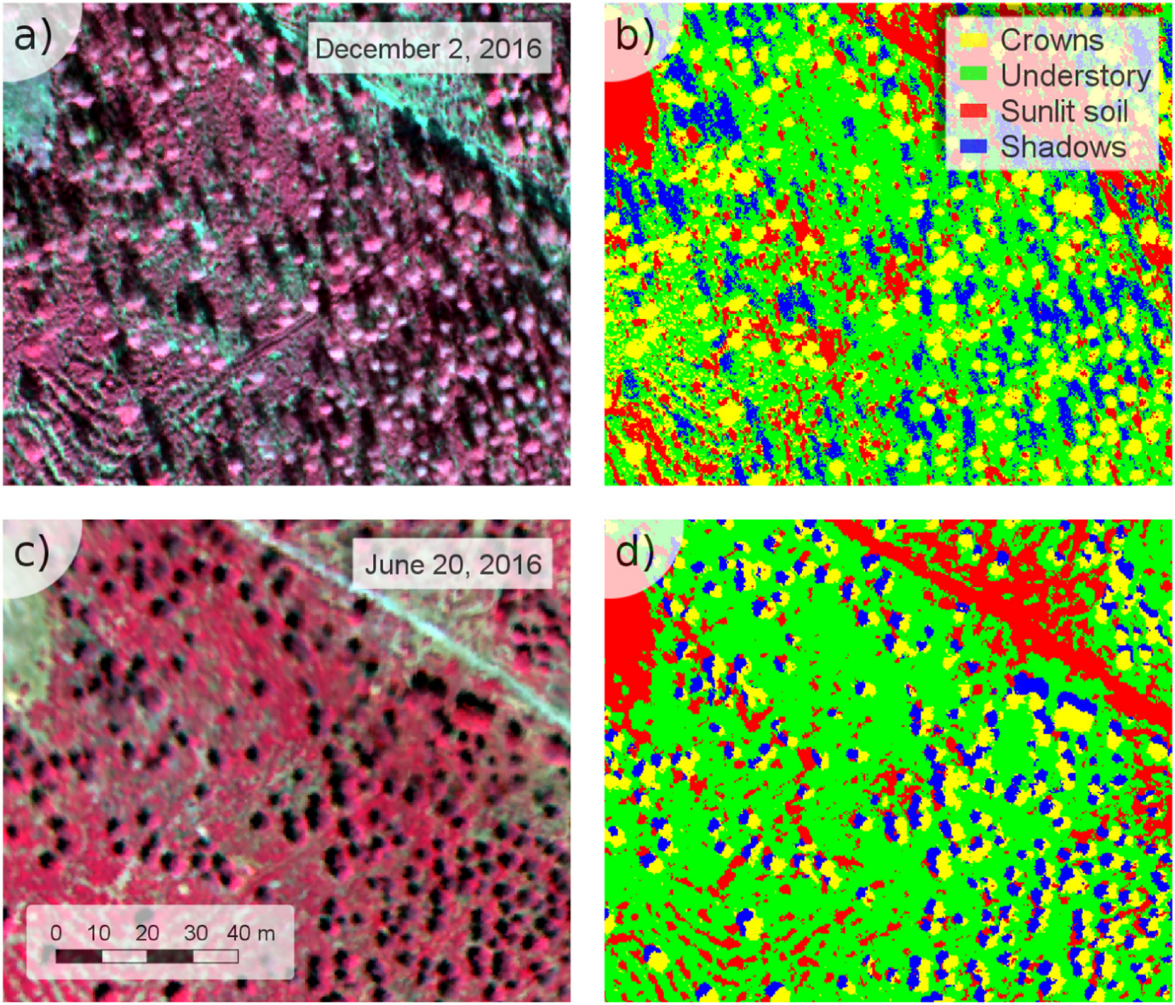
Classification conducted on the December (a, b) and June (c, d) hyperspectral images to generate the four scene components: i) pure tree crowns, ii) understory vegetation, iii) sunlit soil, and iv) shadows.

Object-based segmentation methods were applied to the airborne hyperspectral images using Niblack's thresholding (Niblack, 1986) and Sauvola's binarization techniques (Sauvola and Pietikäinen, 2000) to separate tree crowns from the soil. Overlapping crowns were separated using binary watershed analysis and the Euclidian distance for each object (Fig. 3). The object-based analysis successfully separated vegetated from non-vegetated objects (Fig. 3b) but was unable to distinguish trees from understory. These elements were separated (Fig. 3c) by intersecting the vegetation objects with a supervised classification conducted on each hyperspectral dataset (Fig. 4). The maximum likelihood supervised classification was carried out to identify pure tree crowns, sunlit soil, understory and tree shadows, obtaining a pixel-based map of each scene component for December (Fig. 4ab) and June dates (Fig. 4cd). The intersection of the pixel-based classification for each scene with the object-based segmentation produced a layer of the pure tree crowns at the object level, discarding the understory and shadow components.

**Fig. 5:**
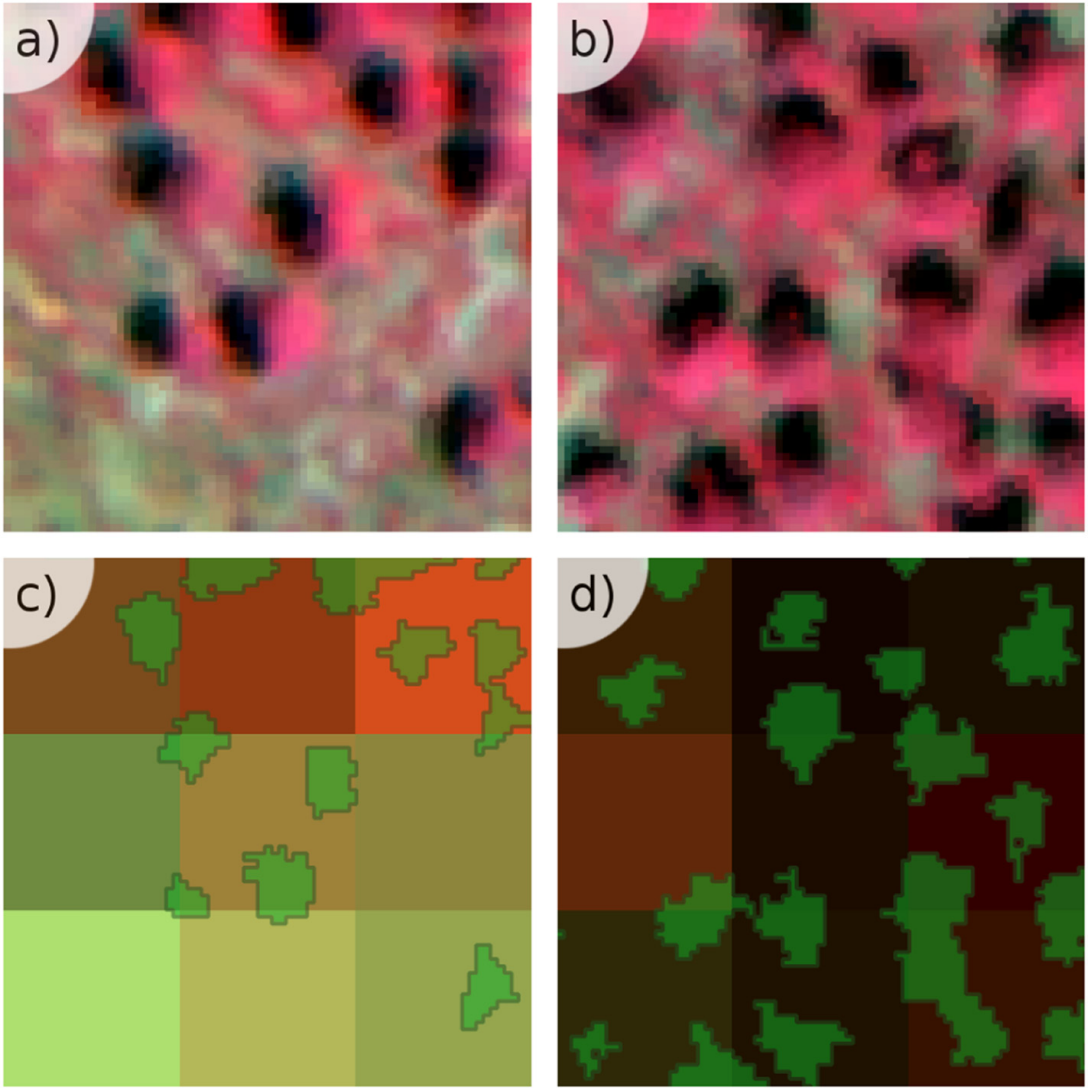
Hyperspectral subsets showing two areas with low (a) and high (b) percentage tree cover. The corresponding Sentinel-2A pixels (c, d) are overlayed on the pure tree crowns obtained through image segmentation.

Following this classification method, the percentage cover and the spectral profile of each scene component were extracted. In particular, the hyperspectral images acquired concurrently with the Sentinel-2A scenes were used to extract the spectra of the individual tree crowns, sunlit understory, shaded understory and sunlit soil that belonged to each of the Sentinel-2 pixels located over each of the 61 study sites. This method enabled the full characterization of all validation sites, as the high-resolution hyperspectral imagery allowed not only the extraction of each scene component spectrum but also the estimation of the percentage cover for each of them, showing a large range of variability within each Sentinel-2A pixel (Fig. 5).

### 2.4 Index-based relationships, scaling up & model inversion for C_a+b_ estimation

From the hyperspectral images, red-edge and NIR indices were calculated from pure-tree crown reflectance. Indices included: i) the red-edge chlorophyll index CI (R_750_/R_710_) (Zarco-Tejada et al., 2001); ii) the TCARI chlorophyll index alone (Haboudane et al., 2002), iii) TCARI normalized by OSAVI (Rondeaux et al., 1996) to minimize changes in LAI (Haboudane et al., 2002); and iv) the Macc index (R_780_-R_710_)/(R_780_-R_680_) designed to increase robustness to directional effects (Maccioni et al., 2001). The chlorophyll CI index based on the 710 nm band was proposed here due to its sensitivity to C_a+b_ while being robust to crown shadows in forest areas, as demonstrated in coniferous forest stands (Zarco-Tejada et al., 2001), and validated later by various other authors (Hernandez-Clemente et al., 2014; Moorthy et al., 2008). Additionally, the red edge CI ratio was also calculated by replacing the 750 nm by the 740 nm band available in the Sentinel-2A bandset.

Crown C_a+b_ per site was determined by model inversion accounting for background understory grasses (as in Darvishzadeh et al., 2008; Si et al., 2012; Kattenborn et al., 2017). In this study, the PROSAIL model that links the leaf reflectance model PROSPECT-D (Féret et al., 2017) and the canopy radiative transfer model 4SAIL (Verhoef et al., 2007), was used in forward mode to generate a look-up table (LUT) consisting of 100.000 simulations. The parametrization of the LUT was based on the inputs described in Table 1. Understory C_a+b_ was estimated by comparing the simulated spectra to the airborne spectra using RMSE as a cost-function, whereas each C_a+b_ estimate was based on the average of the 1000 closest matching LUT entries which were weighted by the RMSE. This hybrid approach enabled the quantification of pixel-level C_a+b_ via a combination of direct validation in the field of the prominent Pinus class, and the quantification of the percentage cover for each scene component within each Sentinel pixel (Pinus trees, understory, shadow and illuminated soil).

**Table 1:** Nominal values and range of parameters used for the C_a+b_ retrieval of the understory vegetation from the hyperspectral imagery.

Canopy structural parameters	Nominal values & range
Leaf area (LAI)	1–4
Average leaf angle (ALA)	30–70°
Hot spot size	0.01


Background & viewing geometry	Nominal values
Soil reflectance (ρ_s_)	From image
Viewing geometry (*θ*_*s*_*θ*_*v*_*ϕ*)	*θ*_*s*_ = 26° (June)/73° (Dec.) *θ*_*v*_ = 0°, *ϕ* = 0°


Leaf parameters	Nominal values
Chlorophyll a + b (C_a+b_)	10–60 μg/cm^2^
Carotenoid content (C_x+c_)	3–12 μg/cm^2^
Anthocyanin content (C_anth_)	0.1–4.0 μg/cm^2^
Dry matter (C_m_)	0.0022 mg/cm^2^
Equivalent water thickness (C_w_)	0.005 mg/cm^2^
Structural parameter (N)	1.5–2.2

C_a+b_ retrieval from Sentinel-2A imagery for each of the study sites was carried out via two approaches: i) using vegetation indices (VIs) proposed in the literature specifically for C_a+b_ estimation using the Sentinel-2A bandset (Clevers and Gitelson, 2013) (Table 2); and ii) via a model-inversion scheme of the Invertible Forest Reflectance Model (INFORM) (Atzberger, 2000; Schlerf and Atzberger, 2012, Schlerf and Atzberger, 2006) based on the Forest Light Interaction Model (FLIM) (Rosema et al., 1992).

**Table 2:** Sentinel-2 chlorophyll indices proposed by Clevers and Gitelson (2013) and used in this study for C_a+b_ estimation in a heterogeneous pine forest.

Index	Formulation	Reference
CI_red-edge_	$\left(\frac{R_{783}}{R_{705}}\right)-1$	Gitelson et al., 2003, Gitelson et al., 2006
CI_green_	$\left(\frac{R_{783}}{R_{560}}\right)-1$	Gitelson et al. (2003, 2006)
REP	$705+35\left(\frac{\left(R_{665}+R_{783}\right)2-R_{705}}{R_{740}-R_{705}}\right)$	Guyot and Baret (1988)
MTCI	$\frac{R_{740}-R_{705}}{R_{705}-R_{665}}$	Dash and Curran (2004)
MCARI/OSAVI_705,750_	$\frac{\left[\left(R_{740}-R_{705}\right)-0.2\left(R_{740}-R_{560}\right)\right]\left(R_{740}-R_{705}\right)}{\left(1+0.16\right)\left(R_{740}-R_{705}\right)/\left(R_{740}+R_{705}+0.16\right)}$	Wu et al. (2008)
TCARI/OSAVI_705,750_	$\frac{3\left[\left(R_{740}-R_{705}\right)-0.2\left(R_{740}-R_{560}\right)\left(R_{740}-R_{705}\right)\right]}{\left(1+0.16\right)\left(R_{740}-R_{705}\right)/\left(R_{740}+R_{705}+0.16\right)}$	Wu et al. (2008)
NDRE1	$\frac{R_{740}-R_{705}}{R_{740}+R_{705}}$	Gitelson and Merzlyak (1994), Sims and Gamon (2002)
NDRE2	$\frac{R_{783}-R_{705}}{R_{783}+R_{705}}$	Barnes et al. (2000)

INFORM was used in forward mode to evaluate the effects of canopy structural parameters on the red-edge bands proposed for C_a+b_ estimation at the Sentinel-2A pixel-scale, in particular, tree density (T_d_), crown diameter (C_d_), and soil reflectance (ρ_s_). For each study site, canopy reflectance was simulated as a function of the percentage cover of each scene component and the mean spectral reflectance for each component extracted from the hyperspectral imagery. Simulated values for canopy reflectance, percentage crown cover and percentage shadow were evaluated against values derived from the high-resolution hyperspectral imagery. Simulated structural NDVI and chlorophyll CI indices were then compared with values derived from the Sentinel-2A and hyperspectral-resampled images to evaluate the performance of the simulation model.

Using 1 million simulations, a LUT was generated using the inputs in Table 3. Leaf parameters dry matter (C_m_), equivalent water thickness (C_w_), and the structural parameter N were estimated using the entire Sentinel-2A bandset, including the SWIR bands at 1610 and 2190 nm. In a second step, with C_m_, C_w_ and N fixed to the retrieved parameters, a second LUT was then generated using the eight VNIR bands. This LUT was used to estimate C_a+b_ based on the set of parameters that minimized the root mean square error (RMSE) for each study site. The estimation of C_a+b_ was carried out for both June and December, using hyperspectral and Sentinel-2A images to evaluate the performance of the model inversion scheme as a function of the sun angle and percentage shadow components for each case.

**Table 3:** Nominal values and range of parameters used for leaf and canopy modelling simulations with INFORM.

Canopy structural parameters	Nominal values & range
Tree density (T_d_)	50–500 trees/ha
Crown diameter (C_d_)	4–5 m
Crown height (C_h_)	7 m
Crown leaf area (C_LAI_)	1–4
Average leaf angle (ALA)	60°


Background & viewing geometry	Nominal values
Soil reflectance (ρ_s_)	From image
Viewing geometry (*θ*_*s*_*θ*_*v*_*ϕ*)	*θ*_*s*_ = 26° (June)/73° (Dec.) *θ*_*v*_ = 0°, *ϕ* = 0°


Leaf parameters	Nominal values
Chlorophyll a + b (C_a+b_)	5–70 μg/cm^2^
Carotenoid content (C_x+c_)	10 μg/cm^2^
Dry matter (C_m_)	0.01–0.035 mg/cm^2^
Equivalent water thickness (C_w_)	0–0.15 mg/cm^2^
Structural Parameter (N)	1.5–2.5

## 3 Results and discussion

### 3.1 Comparison of hyperspectral and Sentinel-2A datasets

**Fig. 6:**
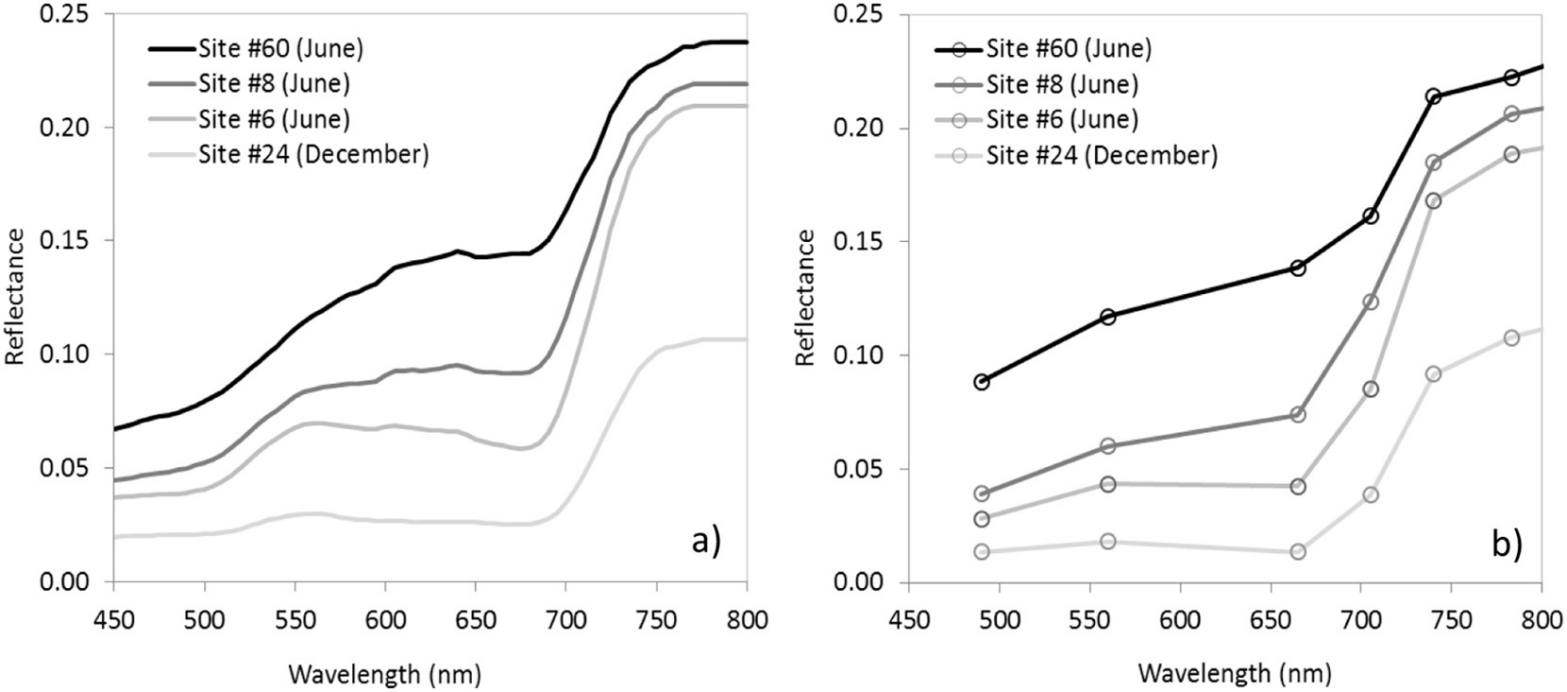
Spatially resampled hyperspectral (a) and Sentinel-2A reflectance (b) from the June and December dates and four validation sites that represented a wide range of variability (Table 4).

Spectral assessment of the hyperspectral mosaics and the Sentinel-2A datasets showed reasonable agreement across the 61 validation sites used in this study (Fig. 6; Table 4). Landscape composition varied widely across the study area, with percentage crown cover ranging between 3% and 34%, understory vegetation cover between 23% and 73%, bare soil cover between 6% and 69%, and shadow cover between 1% and 27%.

**Table 4:** Percentage cover of each scene component from the validation sites displayed in Fig. 6.

Site #	% crowns	% veg. soil	% bare soil	% shadows
6	34.6	32.9	5.9	26.5
8	3.72	73.4	16.7	6.1
60	5.72	23.7	68.9	1.6
24	32.92	26.3	13.4	27.3

**Fig. 7:**
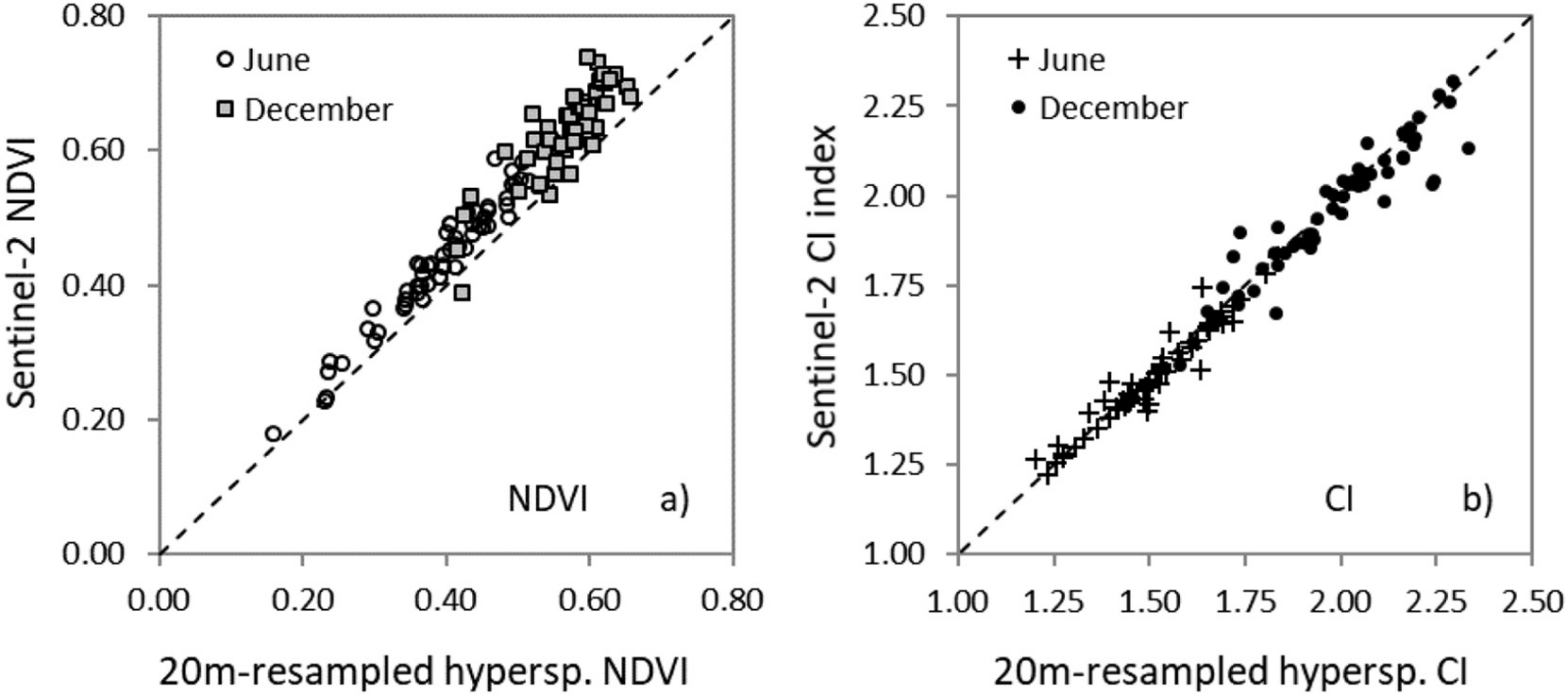
Comparisons carried out for NDVI (a) and CI (b) indices from spatially-resampled hyperspectral vs. Sentinel-2A data for all sites and dates used in this study.

The red and NIR bands (required to calculate NDVI), and the red edge (CI) for both June and December datasets showed good agreement between the 20 m-resampled hyperspectral and the Sentinel-2A data (Fig. 7), with a slight overestimation of NDVI from Sentinel-2A compared to the hyperspectral mosaic (Fig. 7a). Discrepancies between the datasets are potentially related to sensor noise, inaccurate spatial co-registration, or radiometric/atmospheric correction deviations.

### 3.2 Generation of the C_a+b_ dataset from hyperspectral imagery and field measurements as validation for Sentinel-2A

**Fig. 8:**
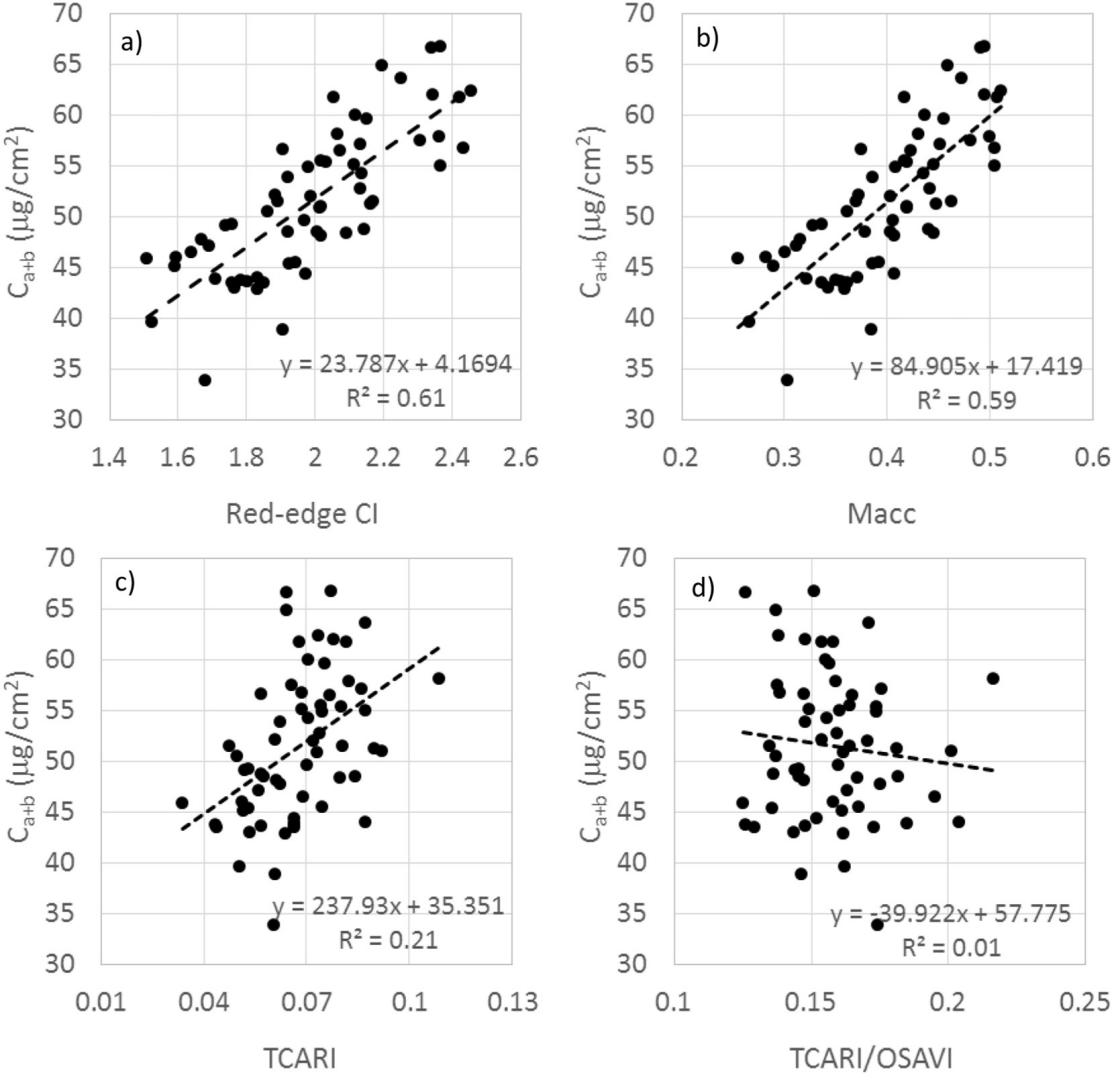
Relationships obtained between the red-edge indices CI (a), Macc (b), TCARI (c) and TCARI/OSAVI (d) calculated from the hyperspectral reflectance of pure tree crowns and the field-measured needle C_a+b_ content.

Of the indices examined (red-edge CI, Macc, TCARI and TCARI/OSAVI), the CI (r^2^ = 0.6; *p* < 0.001) and Macc (r^2^ = 0.59; *p* < 0.001) showed the strongest correlation to field-collected tree-level C_a+b_ estimates, based on pure-crown hyperspectral reflectance (Fig. 8). It is not surprising that the red-edge region outperformed the TCARI and TCARI/OSAVI formulations in this heterogeneous tree crown context, as TCARI was developed for uniform agricultural canopies and the red edge is less sensitive to within-crown shadows (Zarco-Tejada et al., 2001). The results obtained with the red edge CI index using the standard R_750_ band (r^2^ = 0.61) were very similar to the ones obtained with the R_740_ band available in Sentinel-2A (r^2^ = 0.57).

Although reflectance was extracted from pure tree-crown pixels, which effectively removed within-crown shadows (as in Zarco-Tejada et al., 2001), the model inversion conducted by PROSAIL did not yield a statistically significant relationship with field-measured C_a+b_. This was probably due to the improper reflectance simulation of the structural characteristics of pine trees under decline, including dead branches, extensive defoliation and chlorosis. However, the characteristic clumping effects common to coniferous trees may have also accounted for this weak relationship. For this reason, the tree-level empirical relationship between the CI index and field-measured C_a+b_ (Fig. 8a) was used to estimate the C_a+b_ for the Pinus trees falling within each Sentinal-2A pixel at each validation site.

C_a+b_ from each Sentinel-2A pixel was calculated by combining the tree-crown C_a+b_ estimated from the empirical CI index-based relationship (Fig. 8a), with the understory C_a+b_ calculated from the PROSAIL model inversion of the hyperspectral understory component. Table 5 shows the range of C_a+b_ estimates and component percentages from the June and December datasets calculated from the hyperspectral imagery. These hyperspectral-derived C_a+b_ values were used to validate i) Sentinel-2A chlorophyll indices, and ii) the estimation of needle C_a+b_ by INFORM inversion using the radiative transfer modelling method described above.

**Table 5:** Minimum, maximum and average values among the 61 validation sites for the percentage of crowns, understory, bare soil, shadows, crown C_a+b_, understory C_a+b_, and the estimated canopy C_a+b_ content within each Sentinel-2A pixel for June and December datasets.

	Scene Components (%)				June			December		
	%c	%u	%bs	%sh	cC_a+b_	uC_a+b_	pC_a+b_	cC_a+b_	uC_a+b_	pC_a+b_
Min	0.9	5.7	1	0	41.8	16.1	2.4	45.1	16.1	14.2
Max	45.3	80.9	92.6	26.5	52.5	46.9	40.9	69.9	46.9	53.7
Mean	13.5	52.2	27.8	6.6	47.9	40.1	27.6	60.3	40.1	36.7

%c: percentage crowns; %u: percentage understory; %bs: percentage bare soil; %sh: percentage shadows; cC_a+b_: crown chlorophyll *a* + b; uC_a+b_: understory chlorophyll *a* + b; pC_a+b_: Sentinel-2A pixel chlorophyll a + b.

### 3.3 Evaluation of chlorophyll indices from Sentinel-2A imagery

**Fig. 9:**
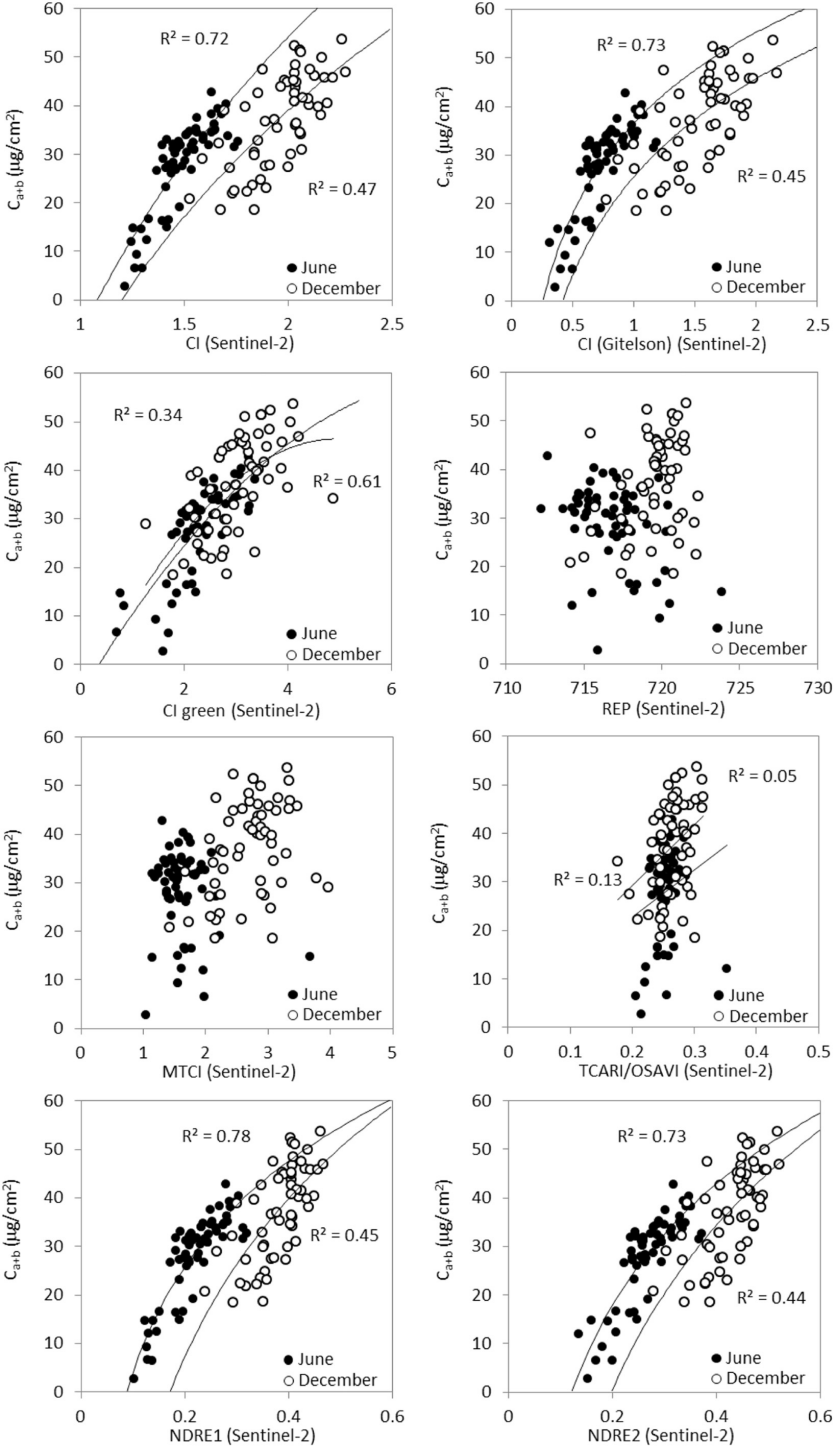
Relationships between Sentinel-2A chlorophyll indices and field-measured needle C_a+b_ for June and December datasets on all 61 validation sites.

Of the chlorophyll indices proposed for Sentinel-2A (Clevers and Gitelson, 2013) (Table 2), the CI, CI-Gitelson, NDRE1 and NDRE2 indices were the most accurate predictors of C_a+b_ (r^2^ > 0.7 for June; r^2^ > 0.44 for December; *p* < 0.001; Fig. 9). Interestingly, the high performance of CI is in agreement with previous studies in Mediterranean pines (Moorthy et al., 2008) and boreal forests (Hernández-Clemente et al., 2012) using hyperspectral and satellite sensors. Most indices had non-linear relationships to C_a+b_, with only the green CI index displaying a more linear trend (r^2^ = 0.34 for June; r^2^ = 0.61 for December; *p* < 0.001). Indices REP, MTCI and TCARI/OSAVI performed poorly for both dates, and except for the green CI, the relationships obtained were displaced as a function of the season.

The displacement observed in the C_a+b_ vs. VI relationships for CI, CI-Gitelson, NDRE1 and NDRE2 indices between June and December (Fig. 9) could be due to different reasons. On the one hand, the phenological changes experienced between summer and winter seasons along with the structural differences may have played an important role in the trends obtained between VIs and C_a+b_. This is particularly relevant due to the large seasonal dynamics of the understory abundance and shadow components in sparse canopies; on the other hand, the seasonal changes in the viewing geometry are known to affect the relationships between VIs and plant traits due to the bi-directional effects of the vegetation canopies, which are increased in the case of sparse canopies. Our results also showed seasonal differences in the needle C_a+b_ concentration measured at the tree crown level (Table 5): in June, the trees displayed lower C_a+b_ content than in December. These changes are potentially associated with seasonal discoloration of Mediterranean species strongly influenced by water stress in summer, in agreement with earlier findings (Camarero et al., 2012; Garcia-Plazaola et al., 1997; García-Plazaola and Becerril, 2001). These reasons justify that the relationships obtained between VIs and plant traits are normally considered to be time-dependent, as they are highly affected by structural, background and viewing geometry effects that are seasonally dependent. Some of these factors may be contributing to the displacements observed in Fig. 9, and in the differences obtained in the goodness of fit between the regression lines obtained in December vs. June. These seasonal changes are normally minimized via radiative transfer models that account for such factors via inversion methods (Thenkabail, 2015).

### 3.4 Structural and background effects on the Sentinel-2A red-edge CI chlorophyll index. Model inversion results for needle C_a+b_ estimation

**Fig. 10:**
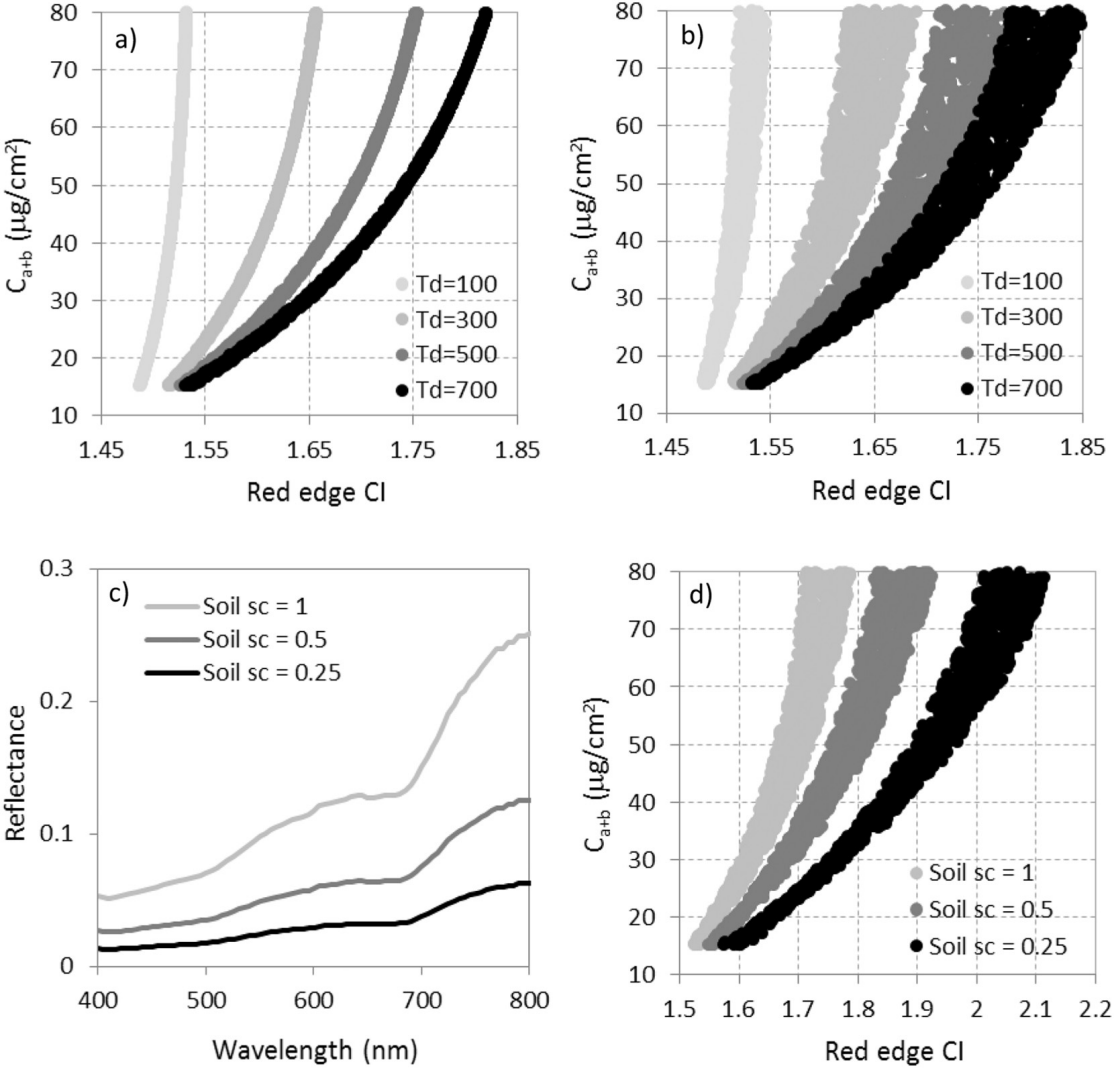
Simulations conducted to display the effects of the tree density (T_d_) variation for fixed (a) and variable crown diameter (C_d_) and tree height (C_h_) (b) on the red-edge chlorophyll index (CI) as a function of needle C_a+b_. For a fixed tree density (T_d_ = 500), the effects on CI as a function of variable soil spectra (c) generated three independent relationships (d) due to the prominent influence of the soil reflectance on open canopies.

Simulations based on the radiative transfer model INFORM showed that the variation of canopy structural variables, i.e. tree density (T_d_), crown diameter (C_d_) and tree height (C_h_) characteristic of open-canopy forest stands have a strong effect on the red-edge chlorophyll index (Fig. 10ab). Soil reflectance was also an important factor, evident in the distinct index-C_a+b_ relationships (Fig. 10d) generated by using three different soil background spectra (Fig. 10c). These results are in agreement with previous studies that used red edge vegetation indices in forest canopies (Hernández-Clemente et al., 2016; Moorthy et al., 2008; Zarco-Tejada et al., 2001). The large effects of background reflectance and tree density are therefore essential for estimating C_a+b_ in an open canopy.

**Fig. 11:**
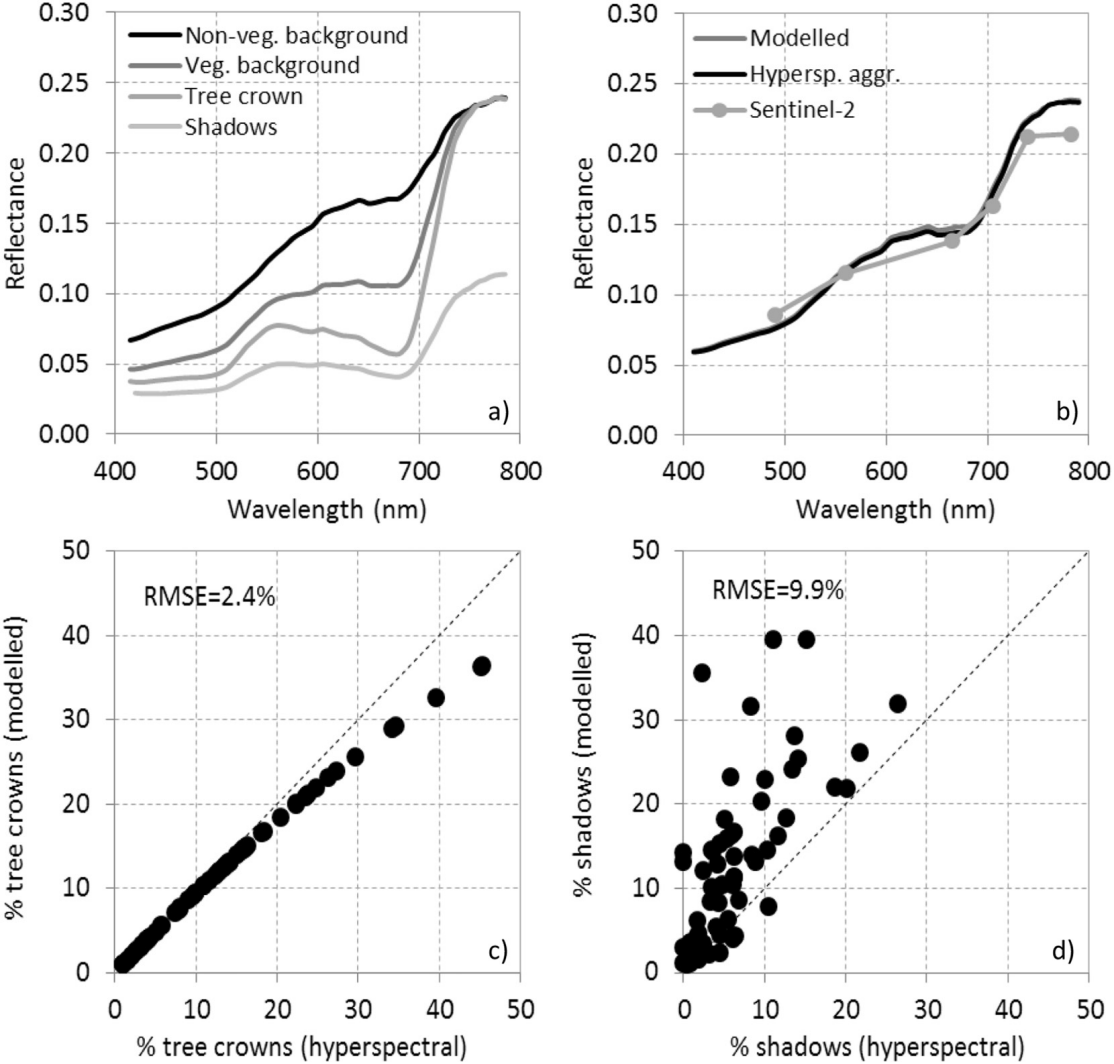
Canopy reflectance simulation conducted using the scene components extracted from the hyperspectral image for one of the validation sites (a) comparing against aggregated hyperspectral and Sentinel-2A imagery (b). The modelled crown percentage cover and the shadow component for all validation sites are shown in (c) and (d), respectively.

Simulations of canopy reflectance from the simple geometrical FLIM model component of INFORM with inputs extracted from the hyperspectral images and their corresponding image-based derived scene components (Fig. 11a) yielded a reasonable agreement (Fig. 11b). Simulations of crown cover (Fig. 11c) and shadow (Fig. 11d) for all validation sites yielded estimations with RMSE = 2.4% (crown cover), with larger errors on the quantification of shadows (9.9%). The errors found in the modelled crown percentage cover (Fig. 11c) were minor as compared to the errors obtained for the shadow component (Fig. 11d). The errors increased when simulating pixels with the crown cover component exceeding 30%, while the model generally overestimated the shadow component even for small proportions. We found out that the quantification of the percentage tree crown component from the hyperspectral imagery was generally easier and more straightforward than the assessment of shadows, particularly under the low sun angles typical of the winter season. For this reason, we cannot point out the model as the only source of error when comparing the simulated scene components vs. the validation dataset. Nevertheless, the RMSE values obtained between the simulations carried out for each study site and the hyperspectral image-based quantification of the scene components were within a reasonable range (2.4% RMSE for the crown component; 9.9% RMSE for the shadow component).

**Fig. 12:**
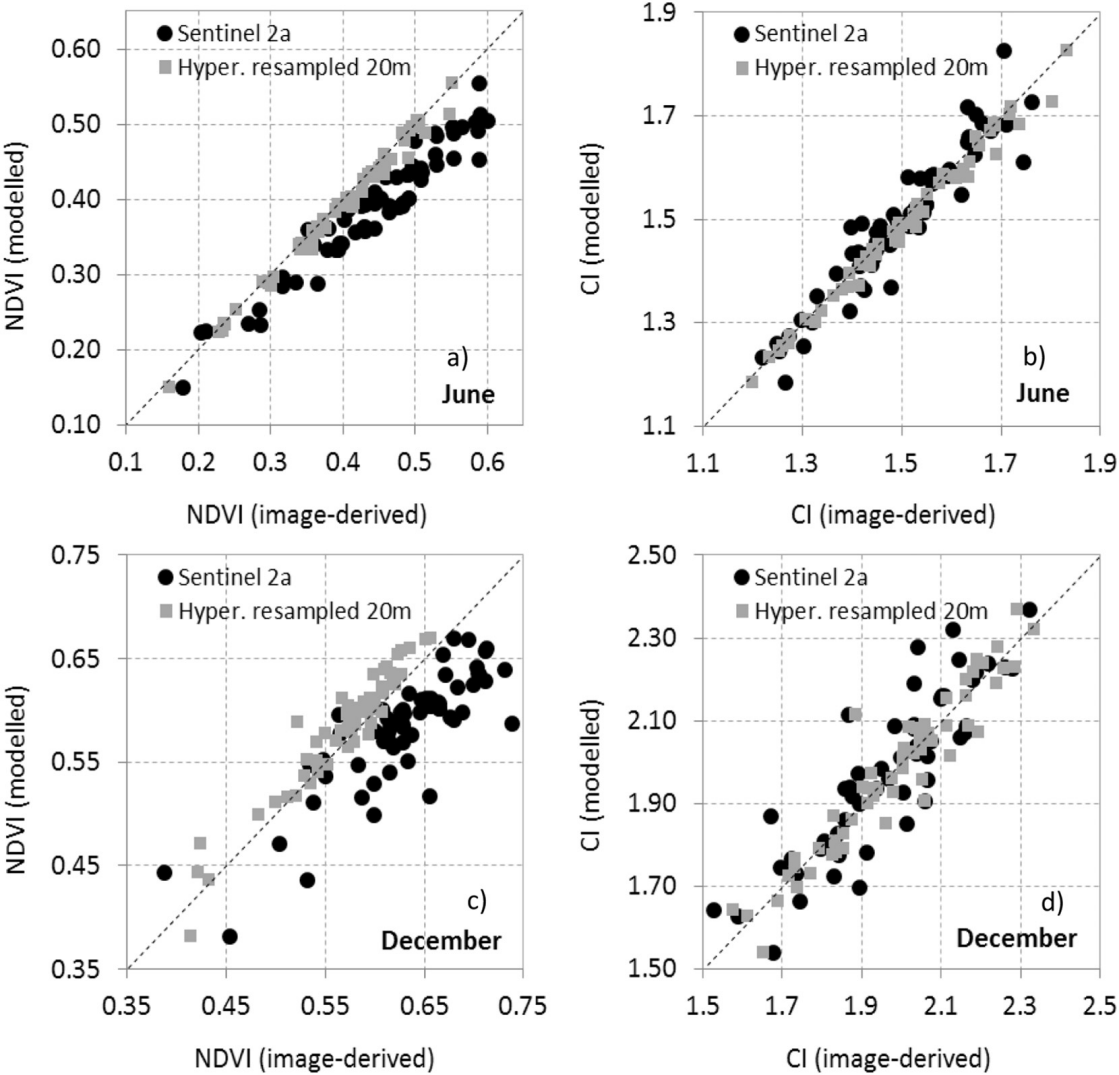
Comparison between NDVI (a, c) and CI (b, d) calculated from resampled hyperspectral imagery and from real Sentinel-2A data for June (a, b) and December (c, d) datasets vs. the modelled validation sites.

NDVI estimates modelled with INFORM in forward mode, using the scene components extracted from the hyperspectral imagery (Fig. 12), were highly correlated (r^2^ > 0.9) with NDVI from resampled hyperspectral data and Sentinel-2A imagery. As expected, the correlation with the resampled hyperspectral images was higher than with the Sentinel-2A imagery. Although the December dataset was more challenging to simulate due to the larger shadow effects (Fig. 12cd), model performance against Sentinel-2A data on the 61 validation sites was reasonable for NDVI (r^2^ > 0.7) and CI (r^2^ > 0.9).

**Fig. 13:**
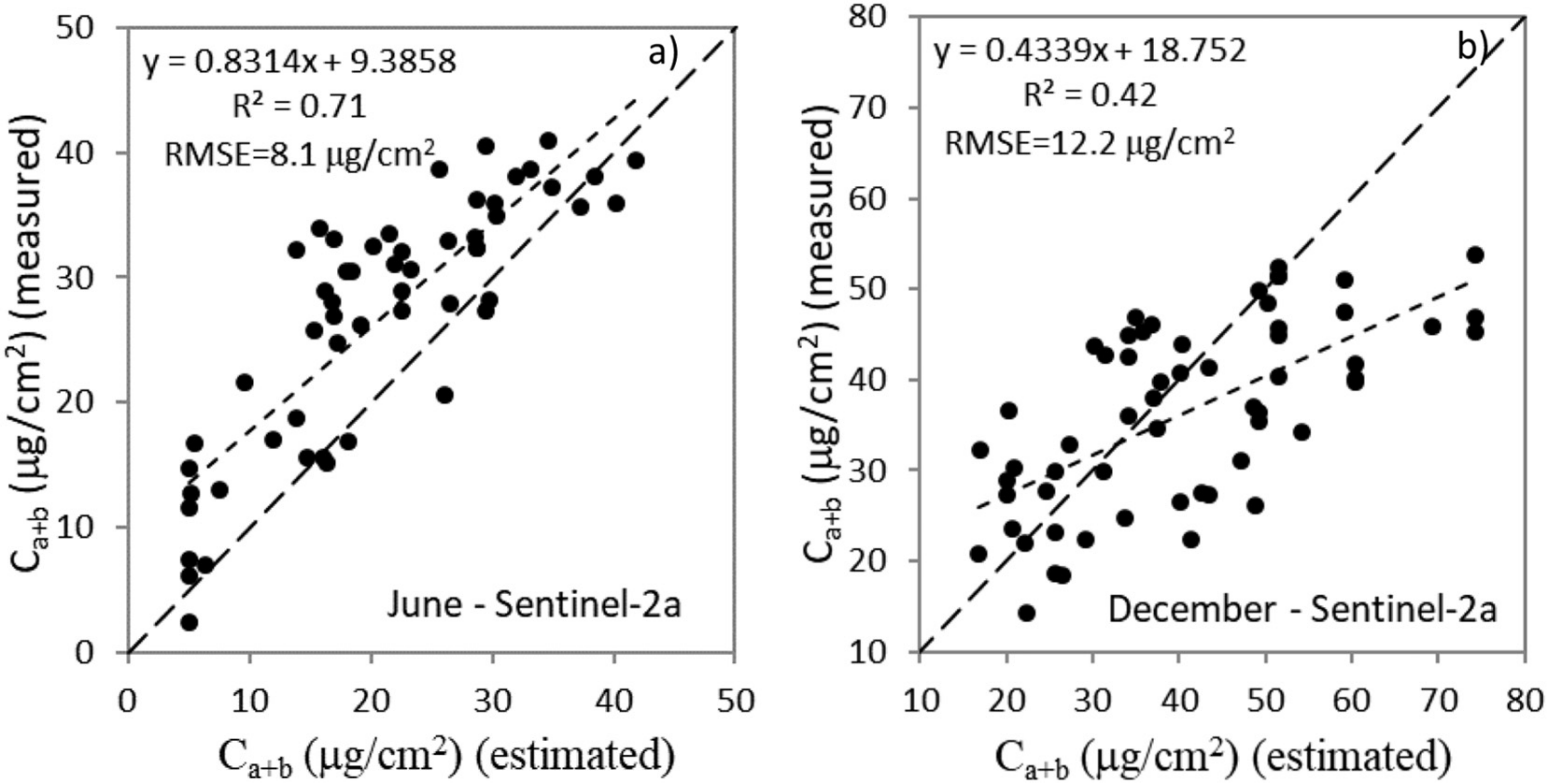
Estimation of needle C_a+b_ by model inversion for June (a) and December (b) using the Sentinel-2A bandset.

The needle C_a+b_ retrieval by model inversion using the LUT generated by INFORM independently for June (Fig. 13a) and December (Fig. 13b) yielded r^2^ = 0.7 (RMSE = 8.1 μg/cm^2^) and r^2^ = 0.4 (RMSE = 12.1 μg/cm^2^), respectively. The overall performance of the model inversion method for the joint dataset (June and December together) yielded r^2^ = 0.6 and RMSE = 10.5 μg/cm^2^. The errors obtained from the Sentinel-2A data for both June and December dates were within the expected RMSE for heterogeneous canopies (i.e. <15 μg/cm^2^). Nevertheless, the higher errors for December compared to June were possibly related with the lower performance of INFORM in simulating the shadow component for scenes with low sun angles, but also to the difficulties in its accurate quantification from the hyperspectral imagery in December.

These results confirm the importance of seasonal phenological activity in the retrieval of chlorophyll content, particularly in sparse canopies. The reduced influence of the understory grasses during spring and summer seem to contribute positively to the performance of the models for the estimation of chlorophyll content. When the same quantification is carried out with data collected during winter, results showed a significant, albeit weaker, model performance. These results could be related to the higher signal-to-noise ratios detected at lower light intensities of mid-latitude winters, the reduced shadow effects and the weaker role played by the understory in summer. The implication of these results arises that spring and summer are the most suitable seasons for quantifying chlorophyll content in conifers in similar conditions and locations.

Although the coefficients of determination for needle C_a+b_ estimation via Sentinel-2A red-edge indices and model inversion methods were similar (r^2^ ~ 0.7), the inversion results had linear relationships with C_a+b_ while the indices had visibly non-linear relationships (Fig. 9). Although index-based methods are useful in that they can be directly applied to the Sentinel-2A images, the complexity of heterogeneous and open-canopy forests require the use of physical models to properly account for canopy structural effects. The Sentinel-2A bandset, which includes red-edge bands, enabled the estimation of needle C_a+b_ only after accounting for the large variations in tree density, and the effects of understory, bare soil and shadows. Our findings have relevance for a considerable portion of the world's forests; our study was based on an open, needle-leaved forest stand with average tree crown cover of 45% (ranging ca 1% and 45%). Globally, 4 million km^2^, or 3% of the Earth's land surface, are covered with coniferous forests within this crown cover range, according to the MOD12Q1 land cover classification following a plant functional type scheme (Friedl et al., 2010; NASA LP DAAC, 2005) and the MOD44B vegetation coniferous fields product (Dimiceli et al., 2015), both produced from MODIS data. In Europe, this comprises 16% of all forests, including Mediterranean forests, as well as boreal forests with a similar architecture. Moreover, Asia and North America, are covered for 4.6 and 5.9%, respectively, by needle-leaved forests with a crown cover between 1 and 45%.

These results highlight the potential of Sentinel-2 for chlorophyll monitoring, especially considering the high spatial and temporal resolution of Sentinel 2A + 2B. Both sensors together will undoubtedly prove useful for ecological and agricultural studies such as disease monitoring, phenology, and primary productivity, theoretically allowing for weekly chlorophyll maps. Although the chlorophyll estimation accuracy was reduced for winter as compared to the summer season, our results showed statistically significant results in both cases. Further work based on complex radiative transfer models yielding superior performance (particularly for the simulation of the viewing geometries typical of winter together with improved modelling of crown shadows in sparse canopies) will potentially improve the prediction performance across seasons.

## 4 Conclusions

We investigated the retrieval of needle chlorophyll content in an open coniferous canopy undergoing decline using Sentinel-2A imagery at two timepoints (June and December) of large sun angle variation. We evaluated the accuracy of needle C_a+b_ estimates from (a) spectral-based Sentinel-2 chlorophyll indices and (b) a radiative transfer model inversion approach. High-spatial resolution hyperspectral imagery acquired concurrently with the Sentinel-2A overpasses enabled the quantification of the Sentinel-2A sub-pixel scene components used for the validation of the radiative transfer methods. The red-edge CI, CI-Gitelson, NDRE1 and NDRE2 indices (r^2^ > 0.7 for June; r^2^ > 0.4 for December; *p* < 0.001) showed the greatest correspondence to ground-based C_a+b_. The INFORM model inversion scheme yielded r^2^ = 0.71 (RMSE = 8.1 μg/cm^2^) for June, and r^2^ = 0.42 (RMSE = 12.2 μg/cm^2^) for December using the entire Sentinel-2A bandset, yielding r^2^ = 0.6 and RMSE = 10.5 μg/cm^2^ for the overall June and December dataset.

Although the coefficients of determination obtained for needle C_a+b_ estimation via red-edge indices and by model inversion were similar (r^2^ ~ 0.7), the model inversion method displayed a linear relationship with C_a+b_, while the relationships for the indices were nonlinear. In these complex forest canopies, accounting for the structure and the variability of each scene component is critical for accurate estimations of C_a+b_. This work demonstrates that C_a+b_ estimation is feasible using Sentinel-2A data across sparse forest canopies, provided this information is available. The present study highlights the potential of Sentinel-2A and 2B for high spatial and temporal resolution chlorophyll monitoring. Both sensors together will evolve as useful tools for vegetation monitoring, producing weekly chlorophyll maps that will improve our understanding of photosynthetic status, physiological condition, and detection of decline processes.
